# The Intestine Plays a Substantial Role in Human Vitamin B6 Metabolism: A Caco-2 Cell Model

**DOI:** 10.1371/journal.pone.0054113

**Published:** 2013-01-14

**Authors:** Monique Albersen, Marjolein Bosma, Nine V. V. A. M. Knoers, Berna H. B. de Ruiter, Eugène F. Diekman, Jessica de Ruijter, Wouter F. Visser, Tom J. de Koning, Nanda M. Verhoeven-Duif

**Affiliations:** 1 Department of Medical Genetics, University Medical Center, Utrecht, Utrecht, The Netherlands; 2 Department of Genetics, University Medical Center, Groningen, University of Groningen, Groningen, The Netherlands; Omaha Veterans Affairs Medical Center, United States of America

## Abstract

**Background:**

Vitamin B6 is present in various forms (vitamers) in the diet that need to be metabolized to pyridoxal phosphate (PLP), the active cofactor form of vitamin B6. In literature, the liver has been reported to be the major site for this conversion, whereas the exact role of the intestine remains to be elucidated.

**Objective:**

To gain insight into the role of the intestine in human vitamin B6 metabolism.

**Materials and Methods:**

Expression of the enzymes pyridoxal kinase (PK), pyridox(am)ine phosphate oxidase (PNPO) and PLP-phosphatase was determined in Caco-2 cells and in lysates of human intestine. Vitamin B6 uptake, conversion and excretion were studied in polarized Caco-2 cell monolayers. B6 vitamer concentrations (pyridoxine (PN), pyridoxal (PL), PLP, pyridoxamine (PM), pyridoxamine phosphate (PMP)) and pyridoxic acid (PA) were quantified by ultra performance liquid chromatography-tandem mass spectrometry (UPLC-MS/MS) using stable isotope-labeled internal standards.

**Results:**

The enzymatic system involved in vitamin B6 metabolism (PK, PNPO and PLP-phosphatase) is fully expressed in Caco-2 cells as well as in human intestine. We show uptake of PN, PM and PL by Caco-2 cells, conversion of PN and PM into PL and excretion of all three unphosphorylated B6 vitamers.

**Conclusion:**

We demonstrate, in a Caco-2 cell model, that the intestine plays a substantial role in human vitamin B6 metabolism.

## Introduction

Vitamin B6 is present in a wide variety of foods, like meat, fish, milk products, potatoes, beans, nuts and several fruits and vegetables [1]. In animal products, it is primarily found as pyridoxal phosphate (PLP) and pyridoxamine phosphate (PMP), whereas plant-derived products mostly contain pyridoxine (phosphate) (PN(P)). PN is widely used as a food supplement [1].

The phosphorylated B6 vitamers are hydrolysed prior to uptake, which takes place in the intestine. In the past, many studies have been published which concluded that vitamin B6 enters intestinal cells by passive diffusion [2], [3], [4], [5]. No saturation was observed and it was thought that vitamin B6 was trapped within the cell by phosphorylation and protein binding. In 2003, a carrier-mediated mechanism for PN uptake in human intestinal epithelial Caco-2 (colorectal adenocarcinoma) cells was reported [6]. Uptake was inhibited by pyridoxamine (PM), but not by pyridoxal (PL) or PLP, suggesting that the transporter protein is selective for two of the three unphosphorylated B6 vitamers. In mammalian colonocytes, a PN uptake mechanism with different characteristics and inhibitable by both PM and PL(P) was found [7]. Despite the biochemical characterization of vitamin B6 uptake, vitamin B6 transporter proteins and their encoding genes have not yet been elucidated.

**Figure 1:**
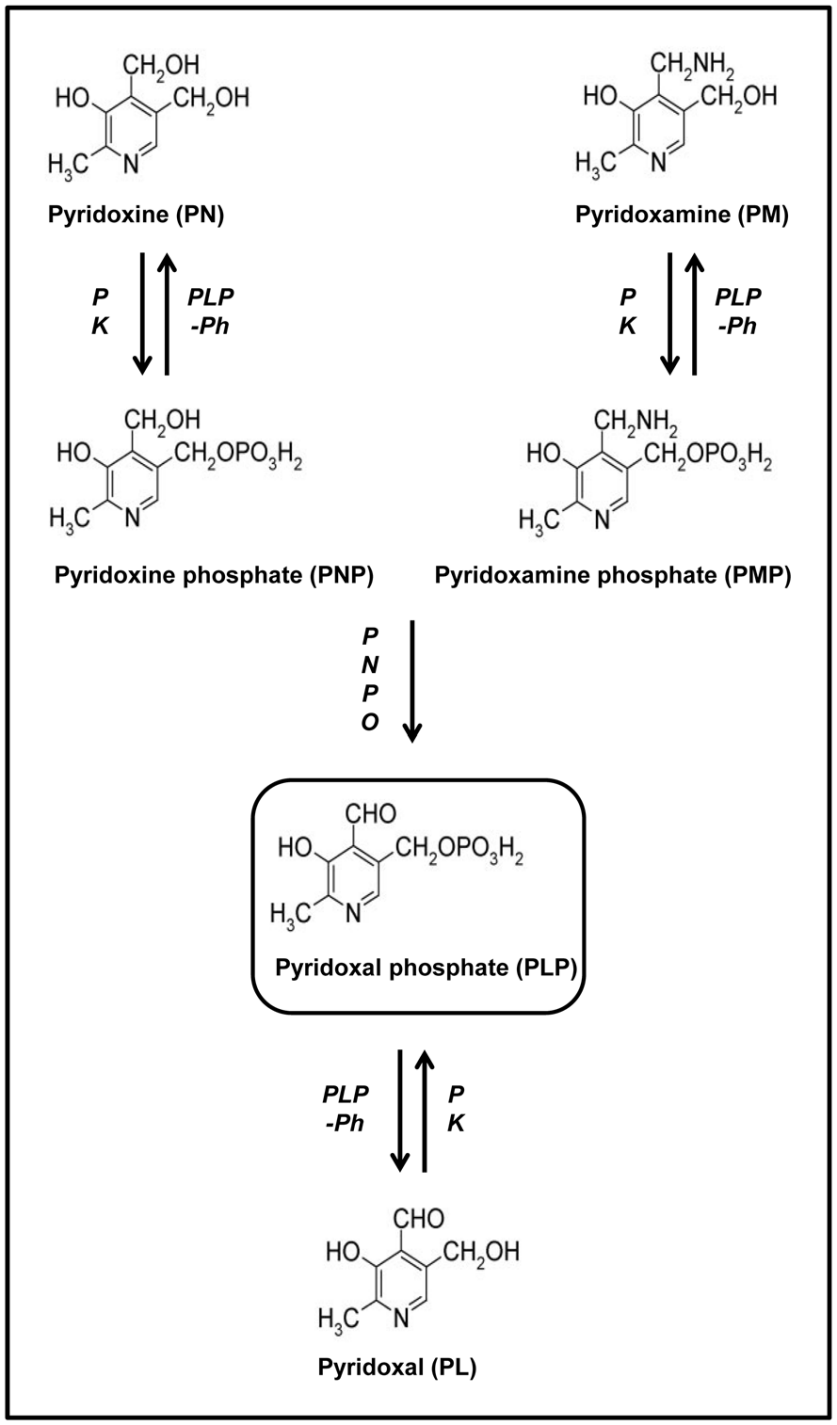
The different vitamin B6 vitamers and their intracellular conversions. PK = pyridoxal kinase. PNPO = pyridox(am)ine phosphate oxidase. PLP-Ph = PLP-phosphatase.

Intracellular vitamin B6 metabolism comprises several steps. First, B6 vitamers are phosphorylated by pyridoxal kinase (PK; EC2.7.1.35). Then, PNP and PMP are oxidized to yield the active form PLP, which is catalyzed by pyridox(am)ine phosphate oxidase (PNPO; EC1.4.3.5). (Figure 1) Hydrolysis of PLP to PL by an intracellular, specific phosphatase (PLP-phosphatase; EC3.1.3.74) [8] and oxidation of PL by pyridoxal oxidase (EC1.2.3.8) [9] constitute the degradation pathway of vitamin B6, of which the major product, pyridoxic acid (PA), is excreted in urine [1]. In plasma, vitamin B6 is present only as PLP and PL [10].

From literature it is not clear whether dietary vitamin B6 is metabolized in the intestine or just taken up by intestinal cells and transported to the liver, where it is metabolized into PLP. The latter hypothesis is supported by many research groups through studies conducted in both humans and rodents. However, this hypothesis is not solidly founded since the intestine was bypassed through intravenous administration of vitamin B6 [11] or intestinal metabolism of B6 vitamers was not at all included in the described experiments [9], [12], [13], [14], [15], [16], [17]. Although it is known that the liver possesses the enzymatic machinery for the formation of PLP from the other B6 vitamers and that liver cells rapidly convert all B6 vitamers into PLP, it has never been convincingly demonstrated that indeed the liver is the main location of PLP formation.

In contrast with these findings are the results of in vivo studies on vitamin B6 metabolism in mice [18], [19], [20]. Trace amounts of [^3^H]-PN and [^3^H]-PM were found to be rapidly absorbed by the intestine. Shortly after administration, only [^3^H]-PL and [^3^H]-PLP were found in the intestine and in portal blood. This suggests that labeled PN and PM are completely converted into [^3^H]-PL and [^3^H]-PLP in the intestine. When ten times higher doses of [^3^H]-PN and [^3^H]-PM were administered, a fraction was released unchanged into the portal blood stream, suggesting that when the maximum capacity of the intestine to convert PN and PM into PL and PLP is exceeded, these B6 vitamers will be excreted by the intestine to be metabolized in the liver. [18], [19], [20].

This discrepancy in literature prompted us to investigate the role of the intestine in human vitamin B6 metabolism in vitro. Caco-2 cells were chosen as a model system for intestinal enterocytes [21] because they can be grown and differentiated into polarized monolayers, creating an apical side representing the intestinal lumen and a basolateral side, corresponding with the portal blood side of the intestine. In this study, we show uptake, conversion and excretion of the unphosphorylated B6 vitamers PN, PM and PL by Caco-2 cells and confirm that all enzymes involved in vitamin B6 metabolism (PK, PNPO and PLP-phosphatase) are present in Caco-2 cells as well as in lysates of human intestine.

## Materials and Methods

### Materials

#### Cell culture

Caco-2 and HepG2 cells were purchased from the ATCC Cell Biology Collection. Dulbecco’s modified Eagle’s medium (DMEM) GlutaMAX-I (20 µmol/L pyridoxine hydrochloride, 4.5 g/L D-Glucose and sodium pyruvate), B6 vitamer-free DMEM GlutaMAX-I (custom made), fetal bovine serum (FBS), penicillin-streptomycin, non-essential amino acids and trypsin-EDTA (0.5%) were purchased from Gibco (Invitrogen Life Technologies). Transwell-COL membrane inserts (12 well cluster, 0.4 µM pore size, 1.12 cm^2^ surface area, PTFE membrane) were purchased from Corning Costar Incorporated.

#### Western Blot

Ethylenediaminetetra-acetic acid (EDTA), sodium dodecyl sulphate (SDS; 10% *w*/*v*) and beta-mercapto-ethanol were purchased from Merck (Schuchardt, Germany). Acetone and sucrose were purchased from Sigma-Aldrich (Steinheim, Germany). Complete protease inhibitor cocktail was purchased from Roche (Woerden, The Netherlands). The bicinchoninic acid (BCA) protein assay kit was purchased from Pierce (Thermo Fisher Scientific Incorporated). Skim milk powder and bovine serum albumin (BSA) were purchased from Fluka Analytical (Sigma-Aldrich). Protran nitrocellulose transfer membranes were purchased from Whatman (Dassel, Germany). Enhanced chemiluminescence (ECL) Western Lightning Plus was purchased from PerkinElmer (Walthan, USA) and high performance chemiluminescence (Hyperfilm ECL) films were purchased from Amersham Biosciences (GE Healthcare).

Human normal whole tissue lysates of small intestine, colon and liver (5.0 mg/mL) in a buffer with protease inhibitor cocktail were purchased from Novus Biologicals. Mouse polyclonal antibodies against partial recombinant PK (H00008566) and PNPO (H00055163) were purchased from Abnova and a mouse monoclonal antibody against PLP-phosphatase (sc-271379) was purchased from Santa Cruz Biotechnology. A secondary goat anti-mouse HRP antibody (32430) was purchased from Pierce. A rabbit antibody against actin (A5060) was purchased from Sigma-Aldrich and a secondary goat anti-rabbit HRP antibody (31460) was purchased from Perbio Science The Netherlands BV (Thermo Fisher Scientific Incorporated).

#### Vitamin B6 metabolism studies and UPLC-MS/MS analysis

Pyridoxine (≥98%), pyridoxal-hydrochloride (≥99%) and pyridoxamine-dihydrochloride (≥98%) were purchased from Sigma-Aldrich. Trichloroacetic acid (TCA, >99%) was purchased from Merck KGaA (Darmstadt, Germany). The internal standards PL-hydrochloride-D_3_ (99%), PN-hydrochloride-^13^C_4_ (99%), PA-D_2_ (98%) and methyl-D_3_-PLP (97%) were purchased from Buchem BV (Apeldoorn, The Netherlands). A Xevo triple quadropole mass spectrometer (TQ MS) with an electrospray ionisation (ESI) source and an Acquity UPLC were used for quantification of B6 vitamers (Waters, Manchester, UK), according to the method described by Van der Ham et al [22].

### Methods

#### Cell culture

Cells were grown and maintained in DMEM GlutaMAX-I supplemented with 10% FBS, 1% penicillin-streptomycin and 1% non-essential amino acids, at 37°C in a humidified atmosphere containing 5% CO_2_. Cells were passed twice a week at >80% confluency by trypsinization with 0.05% trypsin-EDTA after washing twice with phosphate-buffered saline (PBS).

For Western Blot analysis, Caco-2 and HepG2 cells (ATCC Cell Biology Collection) were grown to confluency and Caco-2 cells were additionally differentiated for 14 days. For vitamin B6 metabolism studies, Caco-2 cells from passages 35–40 were seeded on Transwell-COL membrane inserts at a density of 1×10^5^ cells per insert. Membranes were equilibrated for one hour in DMEM GlutaMAX-I with supplements at 37°C before use. Cells were supplied with fresh DMEM GlutaMAX-I medium (with supplements) every three days. Metabolism studies of PN, PM and PL were performed at 14 days of differentiation at 37°C in a humidified atmosphere containing 5% CO_2_.

#### Western Blot

Caco-2 and HepG2 cells were washed twice with room temperature PBS before being harvested with 1.5 mL 0.05% trypsin-EDTA. Trypsin was neutralized by addition of 8.5 mL DMEM GlutaMAX-I medium (with supplements) and supernatants were removed after centrifugation (5 min at 4000 rpm). Cell pellets were washed twice with room temperature PBS and stored at -80°C.

Cell pellets were resuspended in 250 µL of lysisbuffer (50 mM Tris-HCl pH 7.5, 5 mM EDTA, 150 mM NaCl, 10% (*w/v*) sucrose) containing 10 µL of Complete protease inhibitor cocktail. Samples were incubated on ice (10 min), bath-sonificated (15 min) and centrifuged at 4°C (15 min at 13000 rpm). Supernatants were used for determination of protein concentrations using the BCA protein assay kit. Proteins from Caco-2 and HepG2 cell lysates were precipitated with acetone.

Laemmli sample buffer was added to precipitated proteins of Caco-2 and HepG2 cells and to human whole tissue lysates of small intestine, colon and liver (Novus Biologicals). Equal amounts (133 µg) of protein were used. Samples were heated for 5–10 minutes at 95°C. Proteins were separated on a 10% (PK) or 15% (PNPO and PLP-phosphatase) SDS-polyacrylamide gel and subsequently blotted onto a nitrocellulose membrane. Blocking was performed in 5% skim milk.

A two-step incubation with antibody dilutions (for PK and PNPO 1∶1000, for PLP-phosphatase 1∶100 and for anti-mouse HRP 1∶5000 (PK) or 1∶1000 (PNPO and PLP-phosphatase)) in blocking buffer was performed. Actin was used as a loading control (cell lysates only), for which blots were incubated in stripping buffer (100 mM beta-mercapto-ethanol, 2% (*w/v*) SDS, 62.5 mM Tris-HCl pH 6.7) for 30 minutes at 50°C and blocked subsequently in 5% BSA. Again, a two-step incubation with antibody dilutions (for actin 1∶5000 and for anti-rabbit HRP 1∶10.000) was performed. Proteins were visualized on high performance chemiluminescence film using ECL Plus. Western Blotting was performed at least in duplicate for each enzyme.

#### Vitamin B6 metabolism studies and UPLC-MS/MS analysis

Differentiated Caco-2 cell monolayers were washed twice with PBS at room temperature and were pre-incubated for one hour in B6 vitamer-free DMEM GlutaMAX-I medium without FBS, but supplemented with 1% penicillin-streptomycin and 1% non-essential amino acids. A basal B6 vitamer profile of Caco-2 cell monolayers was determined. Uptake, metabolism and excretion of B6 vitamers was studied after addition of different (0, 100 and 1000 nmol/L) concentrations of PN, PM or PL to fresh B6 vitamer-free DMEM GlutaMAX-I medium (with supplements) in the apical compartment (0.5 mL). The basolateral medium (1.5 mL) was replaced by fresh, B6 vitamer-free medium. Apical and basolateral media were collected at 0, 6 and 48 hours. Cells were washed twice with PBS at room temperature and harvested subsequently in 1.5 mL TCA for cell lysis. Cell lysates and media were stored at -80°C until analysis. Experiments were performed in triplicate.

To study potential spontaneous changes in B6 vitamer concentrations in medium, B6 vitamer-free DMEM GlutaMAX-I with supplements and 0, 100 and 1000 nmol/L of either PN, PM or PL was placed at 37°C (humidified atmosphere; 5% CO_2_) and samples were taken at 0, 6 and 48 hours. B6 vitamer concentrations were determined as described below.

Apical, basolateral and intracellular B6 vitamer concentrations (PN, PL(P), PM(P) and PA; nmol/L) were determined by a sensitive and accurate ultra performance liquid chromatography-tandem mass spectrometry (UPLC-MS/MS) method using stable isotope-labeled internal standards [22]. 100 µL of internal standard in TCA was added to 100 µL of medium or cell lysate. After incubation in the dark (15 min at room temperature), samples were centrifuged (5 min at 13000 rpm). 10 µL of the supernatants was used for UPLC-MS/MS analysis of the different B6 vitamers. B6 vitamer concentrations were calculated as the total amount recovered in compartment and cellular fractions (pmol).

B6 vitamer concentrations in medium were corrected for spontaneous changes due to B6 vitamer instability, of which non-enzymatic transamination of PL into PM was most prominent [23].

## Results

### Expression of the Enzymes Involved in Vitamin B6 Metabolism

To investigate whether the enzymes involved in vitamin B6 metabolism (PK, PNPO and PLP-phosphatase) are expressed at protein level in Caco-2 cells and human intestinal whole tissue lysates, Western Blot analysis was performed. Human hepatocellular carcinoma (HepG2) cells and human liver whole tissue lysates were used as positive controls, as high mRNA expression of the enzymes in liver has been reported [24].

**Figure 2:**
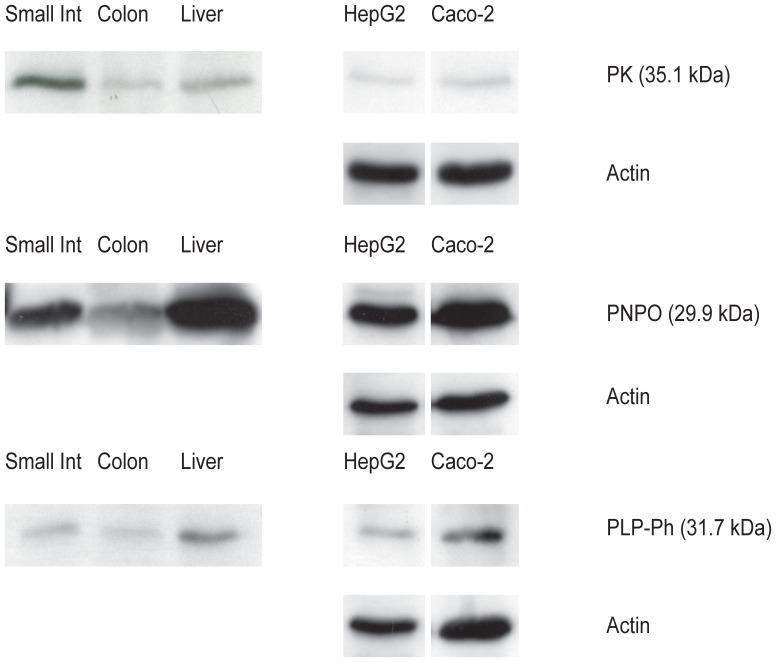
Expression of the enzymes involved in vitamin B6 metabolism in human cell lines and tissue lysates. Actin was used as a loading control (cell lysates only). Depicted are representations of at least duplicates. PK = pyridoxal kinase. PNPO = pyridox(am)ine phosphate oxidase. PLP-Ph = PLP-phosphatase. Small Int = small intestine. HepG2 = HepG2 cells. Caco-2 = Caco-2 cells.

The enzymes PK, PNPO and PLP-phosphatase were present in Caco-2 cells, human whole tissue lysates of small intestine and colon, as well as in HepG2 cells and human liver whole tissue lysates. (Figure 2) All protein bands were visible at the expected heights (for PK: 35.1 kDa, for PNPO: 29.9 kDa and for PLP-phosphatase: 31.7 kDa). In Caco-2 cells, all enzymes were present from one week of differentiation on (data not shown).

### Vitamin B6 Metabolism in Caco-2 Cells

To study uptake of the unphosphorylated B6 vitamers, a basal B6 vitamer profile of differentiated Caco-2 cells was determined after which the monolayers were incubated with 100 and 1000 nmol/L of PN, PM or PL. The metabolic fate of the incubated PN, PM and PL was determined by quantification of all B6 vitamers in apical and basolateral media as well as in cell lysates.

#### Basal B6 vitamer profile of Caco-2 cells

After growth and differentiation in medium containing 20 µmol/L PN, the B6 vitamer profile of Caco-2 cell monolayers was determined. PLP was present in the highest amount (31 pmol), next to PN (24 pmol), PL (19 pmol) and a low amount of PMP (2.3 pmol). Pre-incubation in B6 vitamer-free medium resulted in a release of PN and PL from the Caco-2 cells, but did not alter intracellular amounts of the phosphorylated B6 vitamers PLP and PMP (data not shown). PA was not present intracellularly.

#### Incubation with pyridoxine (PN)

During incubation of the apical side of the Caco-2 cell monolayer with PN, we observed a decrease of apical PN amounts in time, pointing to uptake of PN by Caco-2 cells. In addition, there was a slight increase in basolateral PN amounts during incubation with 1000 nmol/L of PN. (Table 1).

**Table 1:** Apical and basolateral amounts of PN, PM and PL in time during incubation with 100 and 1000 nmol/L of either B6 vitamer.

Amount of B6 vitamer present (pmol )	PN (100 nmol/L added)	PN (1000 nmol/L added)	PM (100 nmol/L added)	PM (1000 nmol/L added)	PL (100 nmol/L added)	PL (1000 nmol/L added)
Apical						
t = 0 hours	50.0	500	50.0	500	50.0	500
t = 6 hours	38.3 (2.4)	429 (23.4)	35.6 (3.5)	397 (25.1)	30.9 (1.8)	320 (8.1)
t = 48 hours	14.3 (1.0)	131 (17.2)	13.4 (0.2)	123 (3.9)	31.2 (0.6)	303 (0.9)
Basolateral						
t = 0 hours	0.0	0.0	0.0	0.0	0.0	0.0
t = 6 hours	1.5 (0.8)	16.2 (4.2)	0.0 (0.0)	9.4 (0.7)	15.4 (2.1)	168 (12.0)
t = 48 hours	0.0 (0.0)	32.7 (2.3)	1.1 (0.2)	31.5 (2.2)	5.4 (2.3)	152 (8.8)

B6 vitamer concentrations were corrected for compartment volume yielding amounts (pmol). Depicted are means (SE) of triplicates.

**Figure 3:**
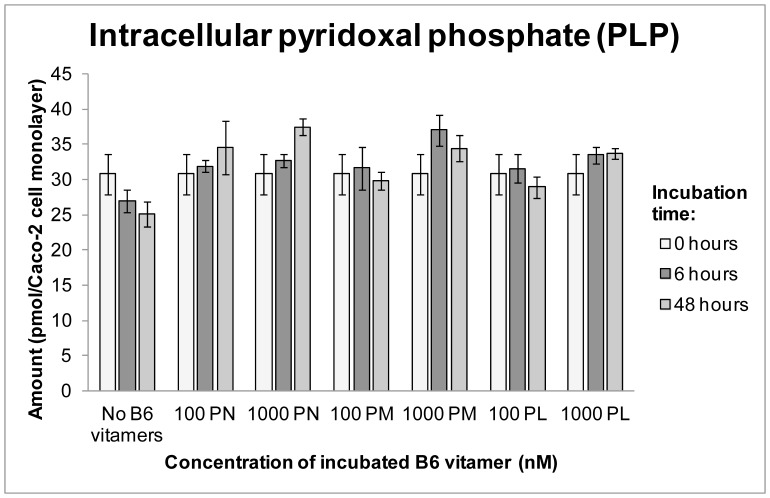
Intracellular amounts of PLP during incubation with no B6 vitamers, PN, PM and PL. Depicted are means ± SE of triplicates.

Metabolic conversions of intracellular PN were studied by looking at changes in amounts of the other B6 vitamers in time. Intracellularly, mainly PLP was present which remained stable during incubation with 100 nmol/L of PN but increased with approximately 23%, from 31±2.9 pmol to 38±1.2 pmol (in 48 hours) during incubation with 1000 nmol/L of PN. (Figure 3) In comparison, during incubation in medium devoid of B6 vitamers, intracellular amounts of PLP decreased with approximately 18%, from 31±2.9 pmol to 25±1.7 pmol. (Figure 3) Intracellular amounts of PMP remained low (2.0±0.3, 2.2±0.3 and 2.7±0.3 pmol at 0, 6 and 48 hours, respectively).

**Figure 4:**
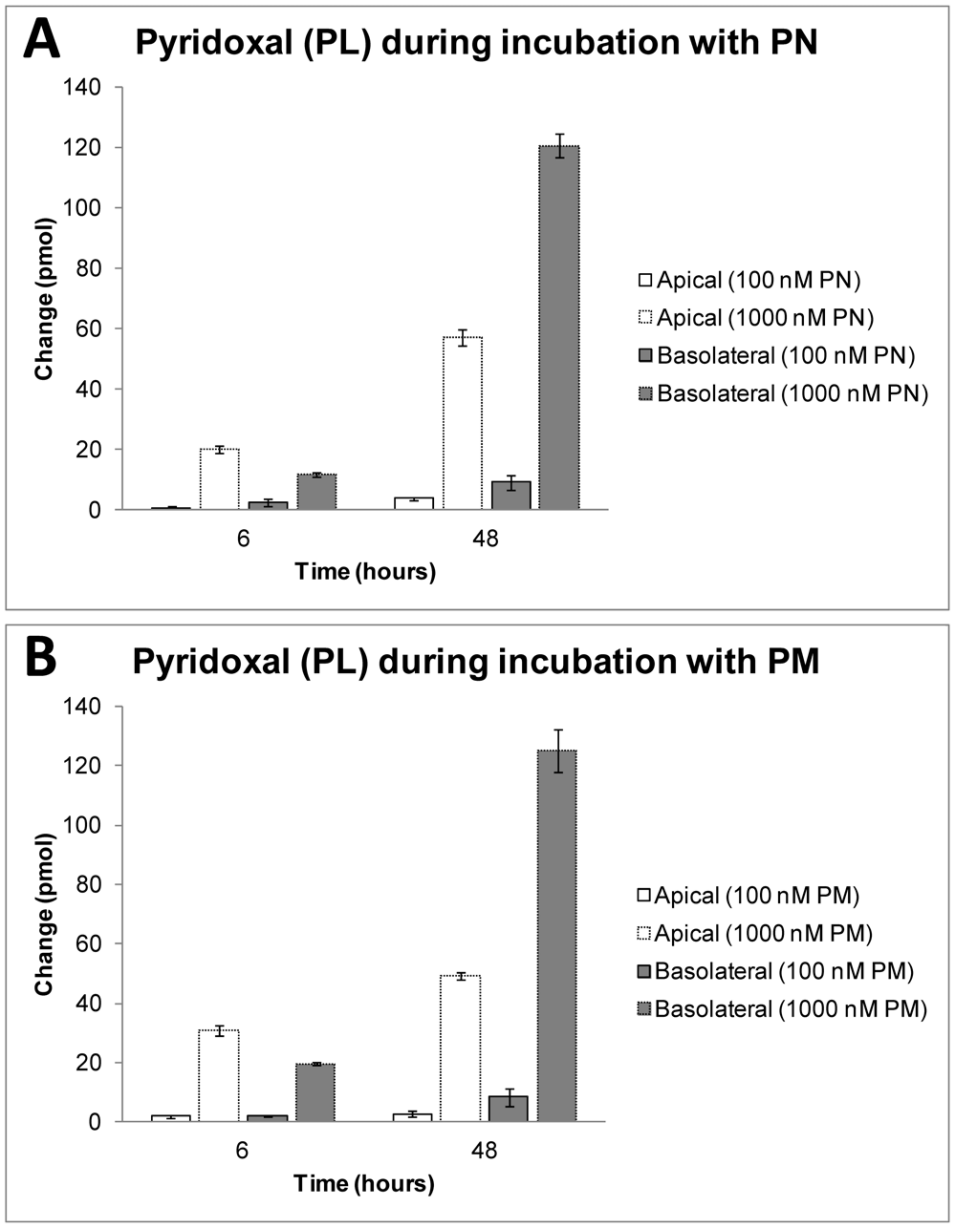
Changes in apical and basolateral amounts of PL during incubation with PN (A) or PM (B). Depicted are means ± SE of triplicates.

In medium, amounts of PL increased in time and with the concentration of incubated PN, demonstrating conversion of PN into PL by Caco-2 cells. Excretion of PL was much higher basolaterally than apically after 48 hours of incubation with PN. (Figure 4A) Amounts of PM did not change in medium during incubation with PN (data not shown). PMP and PLP were absent both apically and basolaterally.

#### Incubation with pyridoxamine (PM)

Results of the experiments in which we incubated with PM were remarkably similar to those in which we incubated with PN. Amounts of PM decreased at the apical side of the Caco-2 cell monolayer in time and increased to some extent at the basolateral side during incubation with high PM concentrations. (Table 1).

Intracellular PLP amounts increased only very slightly during incubation with high PM concentrations. (Figure 3) Intracellular amounts of PMP remained low.

Conversion of PM into PL by Caco-2 cells was obvious from the concentration-dependent excretion of PL in medium, which was higher basolaterally than apically. (Figure 4B) Amounts of PN did not change in medium during incubation with PM (data not shown). PMP and PLP were absent both apically and basolaterally.

#### Incubation with pyridoxal (PL)

Results of incubation of the apical side of the Caco-2 cell monolayer with PL were different from those experiments in which we incubated with PN and PM. The decrease of apical PL amounts in time was only approximately 50% of the decrease found during incubation with PN and PM, suggesting that uptake of PL was less efficient. (Table 1) Furthermore, PL excretion at the basolateral side was approximately equal to PL uptake from the apical side of the Caco-2 cell monolayer. (Table 1).

Like in the experiments with PN and PM, mainly PLP was present intracellularly during incubation with PL and although amounts remained quite stable, they were approximately 16% higher during incubation with 1000 nmol/L compared to 100 nmol/L of PL. (Figure 3) Intracellular amounts of PMP remained low.

Furthermore, no changes in apical and basolateral amounts of PN and PM occurred during incubation with PL (data not shown), suggesting that PL is not converted into the other unphosphorylated B6 vitamers by Caco-2 cells. This is in line with the observed basolateral excretion of PL. PMP and PLP were absent both apically and basolaterally.

#### Formation of pyridoxic acid (PA)

Degradation of the B6 vitamers was studied by quantification of PA in apical and basolateral media as well as intracellularly. Small amounts of PA were present only in the apical medium after 48 hours of incubation with 1000 nmol/L of PN (0.9±0.2 pmol), PM (1.2±0.2 pmol) and PL (3.9±0.1 pmol). To confirm this observation, we studied apical PA excretion during incubation with 10.000 nmol/L of PL, which resulted in at least three times higher apical than basolateral amounts of PA (data not shown). Thus, B6 vitamers can be degraded within Caco-2 cells and subsequently excreted mainly at the apical side of the Caco-2 cell monolayer during incubation with high B6 vitamer concentrations, and especially PL.

## Discussion

Vitamin B6 is the term used to indicate a group of unphosphorylated and phosphorylated pyridine compounds that can be enzymatically interconverted. This interconversion is important, since plant-derived foods mostly contain PN(P) whereas the biologically active cofactor is PLP. The organs that are important in this interconversion have not been irrefutably identified.

**Figure 5:**
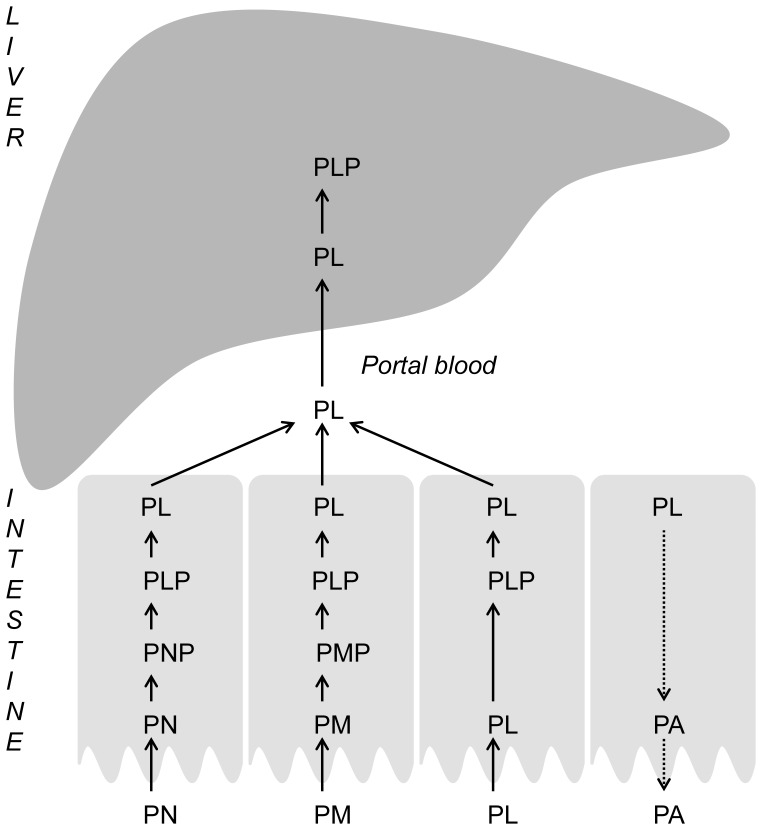
Hypothesis of human intestinal vitamin B6 metabolism.

Here we report studies in a model system of human intestine, using polarized Caco-2-cell monolayers. In this system, we clearly show uptake of PN, PM and PL from the apical incubation medium. PL is not converted into PN or PM, but excreted into the basolateral compartment. In contrast, PN and PM are both converted into PL, probably by the sequential actions of PK (to give PNP and PMP), PNPO (to give PLP) and PLP-phosphatase (to give PL). The formed PL is excreted into the medium, mainly at the basolateral side (basolateral : apical excretion = approximately 2.5∶1), suggesting a PL-specific export system in the basolateral Caco-2 cell membrane. (Figure 5).

Our results are in support of the findings of Sakurai et al [18], [19], [20], who showed uptake of physiological amounts of labeled PN and PM and complete conversion into PL and PLP by the mouse intestine in vivo.

Interestingly, we also observed apical excretion of PA. Previous studies have shown expression of efflux pumps with broad substrate specificity in the apical membrane of polarized Caco-2 cells [25]. Possibly, one of these pumps may be involved in the limitation of transport of PA, the metabolic end product of PLP degradation, from intestinal cells into portal blood. (Figure 5).

In our experiments, PLP was only present intracellularly and amounts changed just to a minor extent during incubation with different concentrations of the unphosphorylated B6 vitamers. Apparently, intracellular levels of PLP are very tightly regulated and PLP is not excreted by Caco-2 cells. This seems in contrast with the studies of Sakurai et al [18], [19], [20] in mice, who detected, next to PL, also PLP in portal blood after administration of PN or PM, suggesting release of both PL and PLP by the intestine. But since PLP was mainly present in the erythrocyte fraction of portal blood, it was concluded that the intestine is able to release only PL, which is converted into PLP in blood. Activity of PK in erythrocytes has been described in literature [26]. In our model system of intestinal enterocytes, the basolateral compartment was used as a reflection of the portal blood side of the intestine, however, without any erythrocytes present. This might explain the absence of PLP in the basolateral medium. (Figure 5).

The enzymes involved in vitamin B6 metabolism, PK, PNPO and PLP-phosphatase, have been reported to be expressed at mRNA level in the liver and to a lesser extent also in the intestine [24]. Expression of PK, PNPO and PLP-phosphatase had not been thoroughly studied, however, at protein level. We show that the enzymatic system involved in vitamin B6 metabolism (PK, PNPO and PLP-phosphatase) is fully present both in Caco-2 cells as well as in human intestinal tissue.

In our Caco-2 cell model, basolateral PN and PM excretion only occurred in case of high apical concentrations of these B6 vitamers. Likewise, in the in vivo studies of Sakurai et al [18], [19], [20], only high doses of PN and PM resulted in the appearance of these B6 vitamers in portal blood. These results suggest that when the maximum capacity of the intestine to convert PN and PM into PL is exceeded, PN and PM will enter the portal circulation and will be metabolized in the liver as well.

Thus, under normal dietary circumstances, it is very likely that not PN, but mainly PL reaches the portal circulation. All other organs, including liver and brain, obtain PL from blood and only need the enzyme PK to form PLP. This suggests that in most tissues, including brain, PNPO is not needed for PLP formation, but that it acts as a recycling enzyme in the salvage pathway of PLP rather than as a PLP synthesis enzyme.

In contrast, when high amounts of PN are administered, the capacity of the intestine is insufficient to fully metabolize all PN. Then, PN may reach the circulation and other tissues. Indeed, in plasma of subjects receiving PN supplementation, we and others detected PN in quantifiable amounts whereas it is normally undetectable in plasma (unpublished observations and [27]). It is likely that not all of this PN is metabolized by the liver, because also in cerebrospinal fluid, high concentrations of PN were observed in PN supplemented patients [22]. The consequences of this unphysiological presence of PN in plasma and cerebrospinal fluid are not yet known.

Our results shed new light on human vitamin B6 metabolism, as we demonstrate a substantial role for the intestine herein.

## References

[1] Bender DA (2005) Water-soluble vitamins: Vitamin B6. In: Geissler CA, Powers HJ, editors. Human Nutrition. London, United Kingsom: Elsevier/Churchill Livingstone. 194–196.

[2] Booth CC, Brain MC (1962) The absorption of tritium-labelled pyridoxine hydrochloride in the rat. J Physiol 164: 282–294. 14013832 10.1113/jphysiol.1962.sp007021 PMC1359304

[3] Hamm MW, Mehansho H, Henderson LM (1979) Transport and metabolism of pyridoxamine and pyridoxamine phosphate in the small intestine of the rat. J Nutr 109: 1552–1559. 479950 10.1093/jn/109.9.1552

[4] Mehansho H, Hamm MW, Henderson LM (1979) Transport and metabolism of pyridoxal and pyridoxal phosphate in the small intestine of the rat. J Nutr 109: 1542–1551. 479949 10.1093/jn/109.9.1542

[5] Middleton HM 3^rd^ (1977) Uptake of pyridoxine hydrochloride by the rat jejunal mucosa in vitro. J Nutr 107: 126–131. 833673 10.1093/jn/107.1.126

[6] Said HM, Ortiz A, Ma TY (2003) A carrier-mediated mechanism for pyridoxine uptake by human intestinal epithelial Caco-2 cells: regulation by a PKA-mediated pathway. Am J Physiol Cell Physiol 285: C1219–C1225. 12867360 10.1152/ajpcell.00204.2003

[7] Said ZM, Subramanian VS, Vaziri ND, Said HM (2008) Pyridoxine uptake by colonocytes: a specific and regulated carrier-mediated process. Am J Physiol Cell Physiol 294: C1192–C1197. 18353902 10.1152/ajpcell.00015.2008

[8] Jang YM, Kim DW, Kang TC, Won MH, Baek NI, et al (2003) Human pyridoxal phosphatase. Molecular cloning, functional expression, and tissue distribution. J Biol Chem 278: 50040–50046. 14522954 10.1074/jbc.M309619200

[9] Merrill AH Jr, Henderson JM, Wang E, McDonald BW, Millikan WJ (1984) Metabolism of vitamin B-6 by human liver. J Nutr 114: 1664–1674. 6088736 10.1093/jn/114.9.1664

[10] Midttun Ø, Hustad S, Ueland PM (2009) Quantitative profiling of biomarkers related to B-vitamin status, tryptophan metabolism and inflammation in human plasma by liquid chromatography/tandem mass spectrometry. Rapid Comm Mass Spectrom 23: 1371–1379. 10.1002/rcm.4013 19337982

[11] Lumeng L, Brashear RE, Li TK (1974) Pyridoxal 5′-phosphate in plasma: source, protein-binding, and cellular transport. J Lab Clin Med 84: 334–343. 4853981

[12] Colombini CE, McCoy EE (1970) Vitamin B6 metabolism. The utilization of [14C]pyridoxine by the normal mouse. Biochemistry 9: 533–538. 5415960 10.1021/bi00805a012

[13] Contractor SF, Shane B (1971) Metabolism of [14C]pyridoxol in the pregnant rat. Biochim Biophys Acta 230: 127–136. 5103256 10.1016/0304-4165(71)90060-2

[14] Johansson S, Lindstedt S, Tiselius HG (1968) Metabolism of [3H8]pyridoxine in mice. Biochemistry 7: 2327–2332. 5660057 10.1021/bi00846a039

[15] Johansson S, Lindstedt S, Tiselius HG (1974) Metabolic interconversions of different forms of vitamin B6. J Biol Chem 249: 6040–6046. 4418204

[16] Lumeng L, Lui A, Li TK (1980) Plasma content of B6 vitamers and its relationship to hepatic vitamin B6 metabolism. J Clin Invest 66: 688–695. 7419716 10.1172/JCI109906 PMC371643

[17] Mehansho H, Buss DD, Hamm MW, Henderson LM (1980) Transport and metabolism of pyridoxine in rat liver. Biochim Biophys Acta 631: 112–123. 7397240 10.1016/0304-4165(80)90059-8

[18] Sakurai T, Asakura T, Matsuda M (1987) Transport and metabolism of pyridoxine and pyridoxal in mice. J Nutr Sci Vitaminol (Tokyo) 33: 11–19. 3612313 10.3177/jnsv.33.11

[19] Sakurai T, Asakura T, Matsuda M (1988) Transport and metabolism of pyridoxine in the intestine of the mouse. J Nutr Sci Vitaminol (Tokyo) 34: 179–187. 3183772 10.3177/jnsv.34.179

[20] Sakurai T, Asakura T, Mizuno A, Matsuda M (1991) Absorption and metabolism of pyridoxamine in mice. I. Pyridoxal as the only form of transport in blood. J Nutr Sci Vitaminol (Tokyo) 37: 341–348. 1765838 10.3177/jnsv.37.341

[21] Halbleib JM, Sääf AM, Brown PO, Nelson WJ (2007) Transcriptional modulation of genes encoding structural characteristics of differentiating enterocytes during development of a polarized epithelium in vitro. Mol Biol Cell 18: 4261–4278. 17699590 10.1091/mbc.E07-04-0308 PMC2043570

[22] Van der Ham M, Albersen M, De Koning TJ, Visser G, Middendorp A, et al (2012) Quantification of vitamin B6 vitamers in human cerebrospinal fluid by ultra performance liquid chromatography – tandem mass spectrometry. Anal Chim Acta 712: 108–114. 22177072 10.1016/j.aca.2011.11.018

[23] Snell EE (1945) The vitamin B6 group. V. The reversible interconversion of pyridoxal and pyridoxamine by transamination reactions. J Am Chem Soc 67: 194–197.

[24] Kang JH, Hong ML, Kim DW, Park J, Kang TC, et al (2004) Genomic organization, tissue distribution and deletion mutation of human pyridoxine 5′-phosphate oxidase. Eur J Biochem 271: 2452–2461. 15182361 10.1111/j.1432-1033.2004.04175.x

[25] Taipalensuu J, Törnblom H, Lindberg G, Einarsson C, Sjöqvist F, et al (2001) Correlation of gene expression of ten drug efflux proteins of the ATP-binding cassette transporter family in normal human jejunum and in human intestinal epithelial Caco-2 cell monolayers. J Pharmacol Exp Ther 299: 164–170. 11561076

[26] Chern CJ, Beutler E (1975) Purification and characterization of human erythrocyte pyridoxine kinase. Clin Chim Acta 61: 353–365. 238769 10.1016/0009-8981(75)90425-8

[27] Footitt EJ, Clayton PT, Mills K, Heales SJ, Neergheen V, et al.. (2012) Measurement of plasma B(6) vitamer profiles in children with inborn errors of vitamin B(6) metabolism using an LC-MS/MS method. J Inherit Metab Dis [Epub ahead of print]. 10.1007/s10545-012-9493-y 22576361

